# Proteolytic maturation of α_2_δ controls the probability of synaptic vesicular release

**DOI:** 10.7554/eLife.37507

**Published:** 2018-06-19

**Authors:** Laurent Ferron, Ivan Kadurin, Annette C Dolphin

**Affiliations:** 1 Department of Neuroscience, Physiology and PharmacologyUniversity College LondonLondonUnited Kingdom; California Institute of TechnologyUnited States

**Keywords:** calcium channel, alpha2Delta subunit, proteolytic processing, synaptic transmission, vesicular release, Rat

## Abstract

Auxiliary α_2_δ subunits are important proteins for trafficking of voltage-gated calcium channels (Ca_V_) at the active zones of synapses. We have previously shown that the post-translational proteolytic cleavage of α_2_δ is essential for their modulatory effects on the trafficking of N-type (Ca_V_2.2) calcium channels (Kadurin et al., 2016). We extend these results here by showing that the probability of presynaptic vesicular release is reduced when an uncleaved α_2_δ is expressed in rat neurons and that this inhibitory effect is reversed when cleavage of α_2_δ is restored. We also show that asynchronous release is influenced by the maturation of α_2_δ−1, highlighting the role of Ca_V_ channels in this component of vesicular release. We present additional evidence that Ca_V_2.2 co-immunoprecipitates preferentially with cleaved wild-type α_2_δ. Our data indicate that the proteolytic maturation increases the association of α_2_δ−1 with Ca_V_ channel complex and is essential for its function on synaptic release.

## Introduction

Among the three families of Ca_V _channels (Ca_V_1, Ca_V_2 and Ca_V_3), the Ca_V_2 family and more specifically Ca_V_2.1 and Ca_V_2.2 channels (generating P/Q and N-type currents, respectively) are particularly important for synaptic transmission in central and peripheral nervous systems (Dolphin, 2012). Ca_V_2.1 and Ca_V_2.2 are targeted to presynaptic terminals where they are responsible for triggering vesicular release (Catterall and Few, 2008; Zamponi et al., 2015). Ca_V_s are formed of several subunits: the α_1_ subunit, that constitutes the Ca^2+^ selective pore and the voltage sensor, and auxiliary subunits β (cytoplasmic) and α_2_δ (extracellular) (Flockerzi et al., 1986; Liu et al., 1996; Takahashi and Catterall, 1987; Witcher et al., 1993). Four genes coding for α_2_δ subunits have been identified (Dolphin, 2012). They are translated into a single pre-protein α_2_δ and post-translationally cleaved into α_2_ and δ peptides, which remain attached by di-sulfide bonds (Dolphin, 2012). In α_2_δ−1 and −2, α_2_ contains a perfect metal ion adhesion site (MIDAS) motif essential for the interaction with α_1_ subunit (Cantí et al., 2005; Hendrich et al., 2008) and δ which is glycophosphatidylinositol (GPI) anchored to the plasma membrane (Davies et al., 2010). The structure of the Ca_V_1.1 channel complex has been recently determined using cryo-electron microscopy and has identified binding domains between Ca_V_1.1 and α_2_δ−1 including the interaction of the α_2_δ MIDAS motif with loop I of the first repeated domain of Ca_V_1.1 (Wu et al., 2016). Site-directed mutagenesis studies have confirmed a functional interaction between α_2_δ−1 and the first extracellular loop of Ca_V_1.2 (Bourdin et al., 2017) and Ca_V_2.2 channels (unpublished results).

α_2_δ subunits are important for the trafficking of α_1_ subunits and their function, and they are also key proteins for synaptic function and synaptogenesis (Dickman et al., 2008; Eroglu et al., 2009; Hoppa et al., 2012; Saheki and Bargmann, 2009; Zamponi et al., 2015). We have recently shown that the proteolytic maturation of α_2_δ−1 into disulfide-linked polypeptides α_2_ and δ is an essential post-translational step enabling its modulatory effect on the activation and trafficking of N-type calcium channels in neurons (Kadurin et al., 2016). Indeed, we show that uncleaved α_2_δ−1 inhibits presynaptic calcium transient-triggered action potential (AP) in hippocampal neurons and that this effect is reversed by the cleavage of α_2_δ−1.

Here, we investigate the impact of the proteolytic maturation of α_2_δ−1 on synaptic release. We used optical tools to measure vesicular release parameters (Ariel and Ryan, 2010; Hoppa et al., 2012). Our data show that an uncleaved α_2_δ−1 reduces the probability of release in response to a single action potential, and also affects asynchronous release. These effects on presynaptic vesicular release are reversed when the cleavage of α_2_δ−1 is restored. We provide additional evidence that cleaved α_2_δ−1 interacts more than the uncleaved form with the Ca_V_2.2 channel pore-forming subunit. Our data indicate that the proteolytic maturation of α_2_δ−1 is important for its association with the Ca_V_ channel complex and its function on synaptic release.

## Results

**Figure 1.**
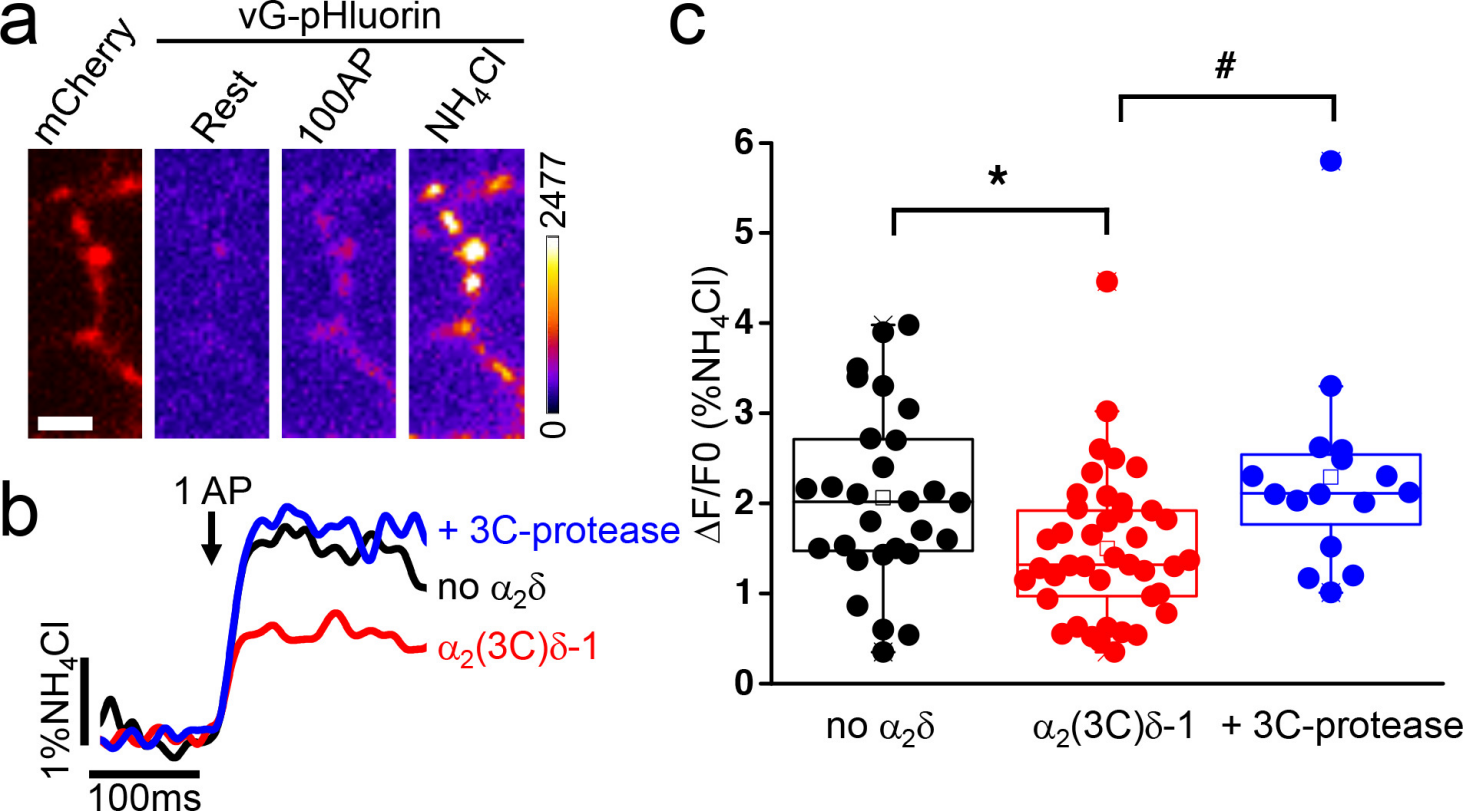
Effect of proteolytic cleavage of α_2_(3C)δ-1 on vesicular release in presynaptic terminals of hippocampal neurons. (**a**) Fluorescence changes in presynaptic terminals of hippocampal neurons expressing vGlut-pHluorin (vG-pHluorin) in response to electrical stimulation. Left panel, mCherry-positive boutons. Three right panels, vG-pHluorin fluorescence: at rest (left), after 100 AP at 10 Hz (middle) and after a brief application of NH_4_Cl (right). Scale bar 5 μm. The pseudocolour scale is shown with the last panel. (**b**) Representative vG-pHluorin responses to a single AP (10–12 trial average, 25 to 50 boutons) from presynaptic terminals of neurons co-transfected with empty vector (black trace), α_2_(3C)δ-1 (red trace) or α_2_(3C)δ-1 + 3C-protease (blue trace). Arrow indicates stimulation with one AP. (**c**) vG-pHluorin fluorescence changes (expressed as % of NH_4_Cl response) in response to 1 AP from boutons co-transfected with empty vector (black), α_2_(3C)δ-1 (red) or α_2_(3C)δ-1 + 3C-protease (blue) (n = 28, 41 and 16 independent experiments, respectively). Box and whiskers plots; *p=0.044 and #p=0.014, one way ANOVA and Bonferroni post-hoc test.

α_2_δ subunits play a crucial role in the trafficking of fully functional calcium channels to the plasma membrane and to presynaptic terminals (Dolphin, 2012). In order to determine the physiological impact of proteolytic maturation of α_2_δ−1, we used the cleavage site mutant α_2_(3C)δ−1 (Kadurin et al., 2016), which is resistant to endogenous proteolysis between α_2_ and δ, to assess the effect of controlled cleavage by exogenous 3C-protease on vesicular release from presynaptic terminals, using the optical reporter vGlut-pHluorin. Transfected hippocampal neurons were identified by mCherry expression (Figure 1a). Neurons were subsequently stimulated (100 AP at 10 Hz), and fluorescence of vGlut-pHluorin was monitored to identify functional boutons (Figure 1a). We first examined the effect of expression of α_2_(3C)δ−1 on synaptic release properties by measuring single AP-evoked exocytosis (Figure 1b). Single AP stimulations were repeated 10 to 12 times with a 45 s rest between each trial. Signals from each bouton were averaged and normalized to the fluorescence value obtained by rapid alkalinization of the entire labeled vesicle pool using NH_4_Cl (Figure 1a and b). Overexpression of uncleaved α_2_(3C)δ−1 induced a decrease of 29 ± 6% in exocytosis compared to the control empty vector condition (n = 28 and 41, respectively; p=0.04) (Figure 1c). Conversely, inducing controlled cleavage of α_2_(3C)δ−1 by co-expressing 3C-protease resulted in an increase of 53 ± 18% in exocytosis compared to α_2_(3C)δ−1 alone (n = 41 and 16, respectively; p=0.014), thus completely reversing the inhibitory effect of uncleaved α_2_(3C)δ−1 (Figure 1b and c).

**Figure 2.**
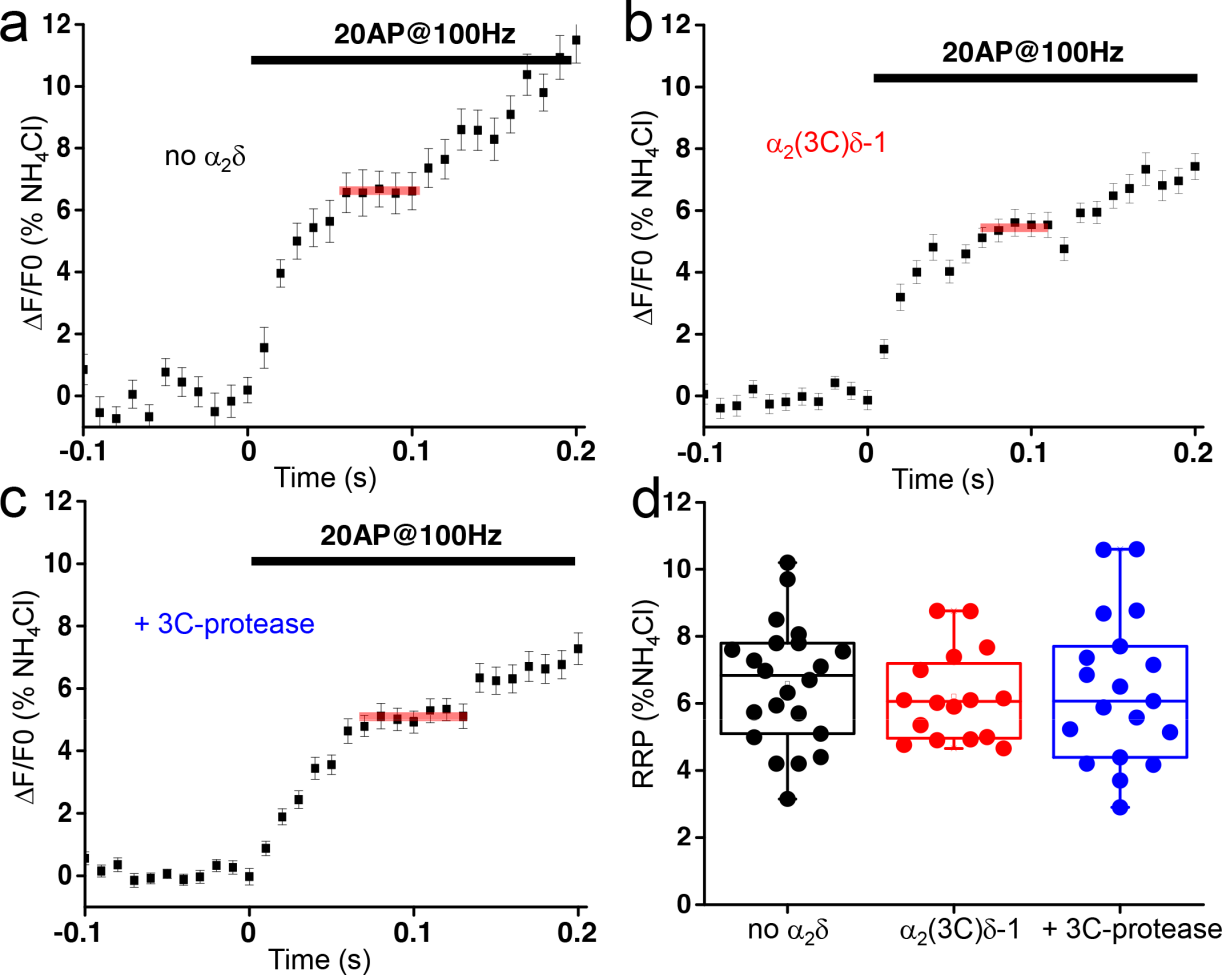
The proteolytic cleavage of α_2_(3C)δ-1 does not affect the readily releasable pool (RRP) in presynaptic terminals of hippocampal neurons. (**a–c**) vG-pHluorin responses (mean ± SEM) to 20 AP at 100 Hz (5–6 trial average, 25 to 50 boutons) from presynaptic terminals of neurons co-transfected with empty vector (**a**), α_2_(3C)δ-1 (**b**) or α_2_(3C)δ-1 + 3C-protease (**c**). Horizontal red lines identify RRPs. (**d**) Average RRP (expressed as % of NH_4_Cl response) from boutons co-transfected with empty vector (black), α_2_(3C)δ-1 (red) or α_2_(3C)δ-1 + 3C-protease (blue) (n = 22, 16 and 19 independent experiments, respectively, p=0.78). Box and whiskers plots; one way ANOVA and Bonferroni post-hoc test.

Synaptic vesicle exocytosis properties are determined by the number of vesicles available for rapid release (the readily-releasable pool - RRP) and the probability (*Pv*) that a vesicle in the RRP will undergo fusion in response to a single AP stimulus (Schneggenburger et al., 2002). RRP can be determined using a high frequency stimulation (Ariel and Ryan, 2010; Ariel et al., 2012). During a 20 AP stimulus at 100 Hz, the fluorescence of vGlut-pHluorin in presynaptic terminals rapidly increases and reaches a plateau phase corresponding to the RRP (Figure 2a). The averaged response, obtained from 5 to 6 trials with a 5 min rest between each trial, were normalized to the size of the total presynaptic pool obtained with NH_4_Cl application (Figure 2a). To examine whether proteolytic maturation of α_2_δ−1 affects the size of the RRP, we imaged neurons transfected with either empty vector (Figure 2a) or α_2_(3C)δ−1 (Figure 2b) or α_2_(3C)δ−1 together with 3C-protease (Figure 2c) and compared the size of the RRP. As summarized in Figure 2d, no difference was recorded between the three conditions (empty vector, α_2_(3C)δ−1 and α_2_(3C)δ−1 with 3C-protease: 6.9 ± 0.4, 6.2 ± 0.3 and 6.4 ± 0.5% of total pool, n = 22, 16 and 19, respectively, p=0.78), indicating that proteolytic maturation of α_2_δ−1 affects the *Pv*, rather than the size of the RRP.

**Figure 3.**
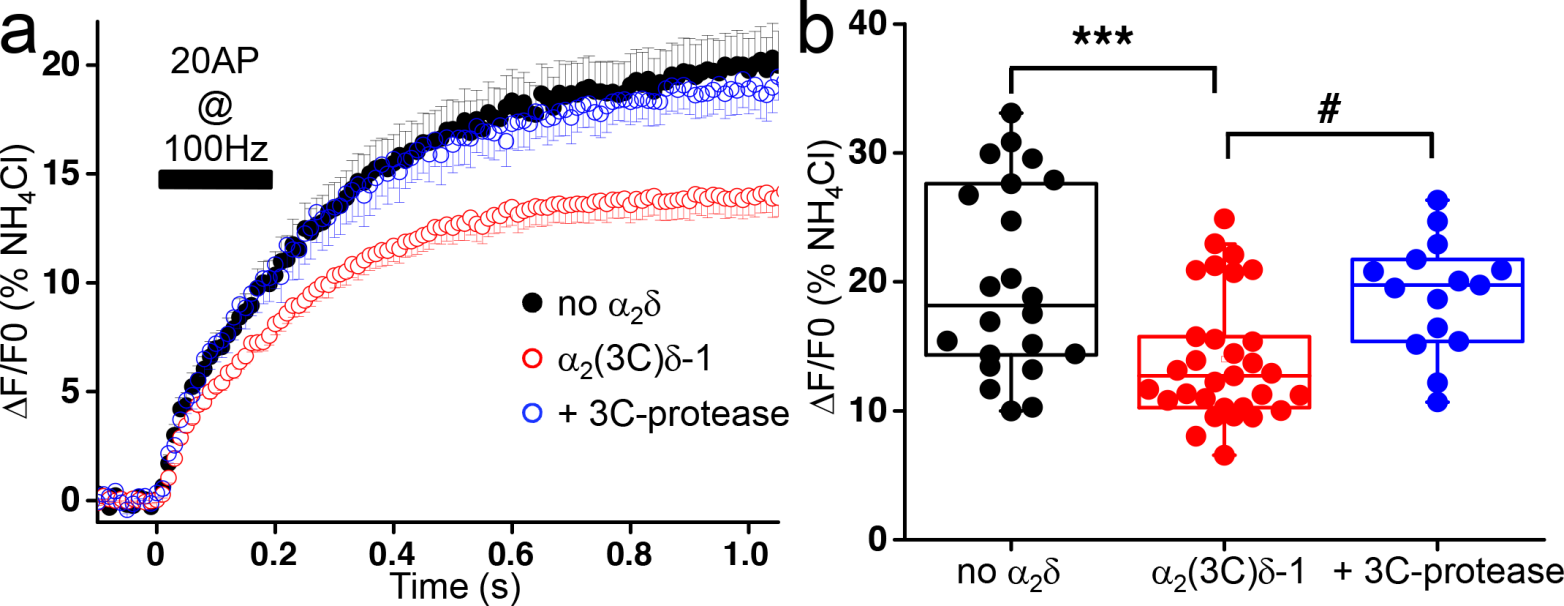
Effect of the proteolytic cleavage of α_2_(3C)δ-1 on delayed vesicular release in presynaptic terminals of hippocampal neurons. (**a**) Average vG-pHluorin responses (mean ± SEM) to 20 APs at 100 Hz (5–6 trial average, 25 to 50 boutons) from presynaptic terminals of neurons co-transfected with empty vector (black), α_2_(3C)δ-1 (red) or α_2_(3C)δ-1 + 3C-protease (blue). The black bar indicates the stimulation period (20 AP at 100 Hz). (**b**) Average delayed vesicular release (expressed as % of NH_4_Cl response) measured 1 s after the beginning of the stimulation from boutons co-transfected with empty vector (black), α_2_(3C)δ-1 (red) or α_2_(3C)δ-1 + 3C-protease (blue) (n = 22, 31 and 15 independent experiments, respectively). Box and whiskers plots with superimposed individual experiments; ***p<0.001 and # p=0.021, one way ANOVA and Bonferroni post-hoc test.

After the plateau phase corresponding to the RRP, an additional increase in fluorescence takes place during the stimulation, and continues for more than 500 ms after the end of the stimulus before reaching a stationary phase (Figure 3a). It was proposed that this secondary increase in fluorescence results from a combination of RRP refilling and slow decay of the elevated intracellular Ca^2+^ concentration (Ariel and Ryan, 2010). This late increase in fluorescence occurs at lower rate than the initial increase and represents post-stimulus exocytosis. Overexpression of uncleaved α_2_(3C)δ−1 induced a decrease of about 30% in this phase of exocytosis compared to control empty vector condition (n = 22 and 31, respectively; p<0.001) (Figure 3b–c). This reduction of delayed exocytosis is completely prevented by the co-expression of α_2_(3C)δ−1 with 3C-protease (Figure 3a–b).

**Figure 4.**
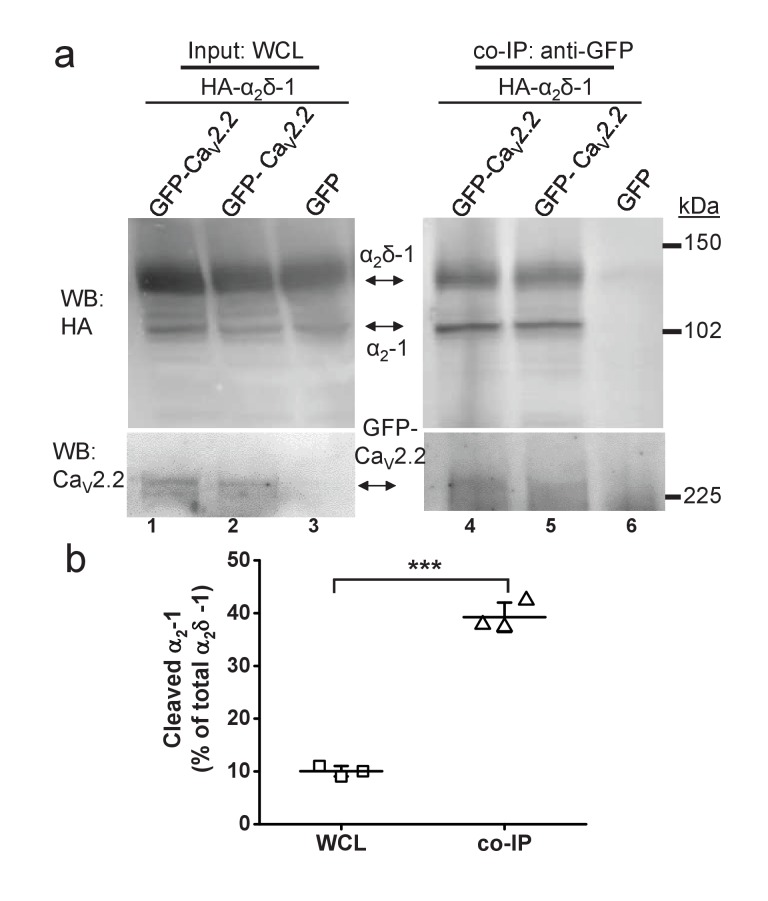
Quantified co-immunoprecipitation of Ca_V_2.2 with cleaved and uncleaved fractions of wild-type α_2_δ-1 from WCL of tsA-201 cells. (**a**) Left panels show WCL input from tsA-201 cells transfected with GFP-Ca_V_2.2 (lanes 1 and 2) or GFP (lane 3), plus β1b and HA-tagged α_2_δ-1: upper panel, HA-α_2_δ-1 input; lower panel, Ca_V_2.2-GFP input. Right panels show immunoprecipitation (IP) of GFP-Ca_V_2.2 with anti-GFP Ab; immunoblots with Ca_V_2.2 II-III loop Ab (lower panels, lanes 4 and 5) produced co-immunoprecipitation (co-IP) of HA-α_2_δ-1 (corresponding upper panels lanes 4 and 5), revealed by anti-HA mAb. All samples deglycosylated. (**b**) Proteolytic cleavage of α_2_δ-1 expressed as percentage of cleaved α_2_-1 moiety to total α_2_δ-1 calculated for input WCL fractions (squares) and for fractions co-immunoprecipitated with GFP- Ca_V_2.2 (triangles). The cleaved α_2_-1 moiety in the co-IP fractions is increased by 29.2 ± 1.7% compared with the WCL fractions (average of 3 independent experiments). ***p=0.0032, paired t-test.

We then wished to determine whether the results obtained on presynaptic release were due to differential interaction of cleaved and uncleaved α_2_δ with the α1 subunit. We have previously shown that transient expression of α_2_δ−1 in cell lines results in only a partial cleavage of wild type α_2_δ−1, such that a mixture of cleaved and uncleaved α_2_δ protein appears in the whole cell lysate (WCL) (Kadurin et al., 2012, 2016). We performed co-immunoprecipitation of wild type α_2_δ−1 with Ca_V_2.2 from tsA-201 cell WCL and found that the percentage of cleaved α_2_δ−1 in the co-immunoprecipitated fractions is ~4 fold higher than the percentage of cleaved α_2_δ−1 in the input WCL (from 10.0 ± 0.6% to 39.2 ± 1.6% in WCL and co-immunoprecipitated fractions, respectively, n = 3) (Figure 4), suggesting stronger association of mature cleaved α_2_δ−1 with the Ca_V_ pore-forming subunit.

## Discussion

Ca_V_2 channels are important for synaptic transmission and their targeting to the active zone is tightly regulated (Catterall and Few, 2008; Simms and Zamponi, 2014). α_2_δ subunits have been shown to control the trafficking of Ca_V_2 to presynaptic terminals (Hoppa et al., 2012). α_2_δ subunits are post-translationally proteolysed, and this process is key for their regulatory action on Ca_V_2 channels (Kadurin et al., 2016). Here, we show that the post-translational proteolytic maturation of α_2_δ−1 is also essential for these proteins to fulfil their regulatory function on vesicular release in presynaptic terminals of hippocampal neurons in culture. Interestingly, we show that both synchronous and asynchronous releases are affected, both release mechanisms being highly dependent on Ca^2+^ influx through Ca_V_2 channels.

Vesicular release is characterized by two key presynaptic parameters: the RRP and *Pv* (Ariel and Ryan, 2012; Schneggenburger et al., 2002). A previous study has shown that over-expression of α_2_δ subunits and knock-down of endogenous α_2_δ increased and decreased *Pv*, respectively (Hoppa et al., 2012). In good agreement with this, our data show that uncleaved α_2_δ−1 (α_2_(3C)δ−1) reduces *Pv*, and co-expression of the 3C-protease restores the control *Pv*. Interestingly, *Pv* is modulated by the number of Ca_V_2 channels in each active zone (Ermolyuk et al., 2012) and we have previously shown that uncleaved α_2_δ subunits reduced the amplitude of calcium transients triggered by a single AP stimulation, by interfering with the trafficking of Ca_V_2 channels (Kadurin et al., 2016). The active zone proteins Rab-3 interacting molecules (RIMs) and Munc-13, critical in the orchestration of synaptic vesicular release, have been shown to control the targeting of Ca_V_2 channels within presynaptic terminals (de Jong et al., 2018; Südhof, 2012). These active zone proteins have also been shown to control the size of the RRP (Augustin et al., 1999; Calloway et al., 2015; Deng et al., 2011; Kaeser et al., 2011). The RRP is defined as a small fraction of vesicles in a presynaptic terminal that is available for immediate release with a brief stimulus train, and thus likely to equate to docked vesicles identified by electron microscopy (Ariel and Ryan, 2012; Rizzoli and Betz, 2005; Schneggenburger et al., 2002). Experimental methods used to estimate the size of the RRP have been recently reviewed and consist of two electrophysiological methods (post synaptic current recordings and presynaptic membrane capacitance measurements) and one optical method (Kaeser and Regehr, 2017). Here, we used the optical technique that was developed by Ariel and Ryan, (2010). This high-time resolution optical method measures exocytosis by detecting fluorescence from pHluorin tagged vGlut-1 (Voglmaier et al., 2006) associated with vesicle fusion. The high frequency stimulation protocol (20 APs at 100 Hz) induces a rapid rise in fluorescence followed by a plateau corresponding to a state during which all the vesicles in the RRP have fused with the membrane. The size of the RRP we describe here, which is determined by the amplitude of the fluorescence of the plateau (6–7% of the total pool of vesicles) is in good agreement with previously described values of RRP in neonatal rodent hippocampal neuron synapses (Ariel and Ryan, 2010; Fernández-Alfonso and Ryan, 2006; Rizzoli and Betz, 2005). A previous study has shown that wild-type α_2_δ subunits have no effect on the size of the RRP (Hoppa et al., 2012). Consistent with that study, our data show that uncleaved α_2_δ−1 does not affect the size of the RRP indicating that, unlike RIMs and Munc13, α_2_δ−1 does not have the same dual function on synaptic vesicular release.

There are two potential mechanisms to account for the reduction in *Pv* by α_2_(3C)δ−1. It is likely that α_2_(3C)δ−1 reduces the trafficking of endogenous Ca_V_2 channels into active zones, as we showed for exogenously expressed Ca_V_2.2 (Kadurin et al., 2016). However, α_2_(3C)δ−1 can also traffic alone into presynaptic terminals (Kadurin et al., 2016), where it could then displace the endogenous α_2_δ interacting with channels in active zones, thus forming non-functional channels. The finding here that uncleaved α_2_δ interacts less than cleaved α_2_δ with Ca_V_2.2 may indicate that the former mechanism is more likely.

Several reports have also described a role for α_2_δ subunits in synaptogenesis, independently from their role as a Ca_V_ auxiliary subunit (Dickman et al., 2008; Eroglu et al., 2009; Kurshan et al., 2009). α_2_δ subunits are extracellular proteins anchored to the plasma membrane via a GPI moiety (Davies et al., 2010) which makes them potentially good candidates to interact with extracellular ligands such as thrombospondins, low density lipoprotein receptor-related protein and α-neurexin (Eroglu et al., 2009; Kadurin et al., 2017; Tong et al., 2017). Although a direct interaction between α_2_δ and thrombospondin and its role in the mediation of synaptogenesis remains controversial (Lana et al., 2016; Xu et al., 2010), altogether these reports suggest that α_2_δ subunits could play a role as an extracellular coordinator of synaptic function. Furthermore, the modulation of presynaptic Ca_V_ channels by proteolytic cleavage of α_2_δ subunits could serve as an additional regulatory mechanism for their complex synaptic functions at the post-translational level.

Ca_V_2 channels and BK potassium channels are known to be part of multi-molecular complexes in neurons (Berkefeld et al., 2006; Müller et al., 2010). α_2_δ−1 has very recently been shown to interact with BK channels, and this interaction was found to reduce the stability of Ca_V_2.2 channels at the plasma membrane by preventing α_2_δ−1 interacting with Ca_V_2.2 channels (Zhang et al., 2018). Functionally, BK channels were shown to control neurotransmitter release by shortening the AP duration and reducing Ca^2+^ influx into presynaptic elements at neuro-muscular junctions (Protti and Uchitel, 1997; Yazejian et al., 1997). Although their presence in presynaptic boutons has been disputed (Hoppa et al., 2014), BK channels are also expressed in axons from central neurons (Debanne et al., 2011; Johnston et al., 2010). Furthermore, α_2_δ−1 has also recently been shown to interact with NMDA glutamate receptors (NMDARs) (Chen et al., 2018), albeit via a C-terminal domain of α_2_δ−1 that is beyond the GPI-anchor attachment site and would therefore not be present in a mature GPI-anchored form (Davies et al., 2010; Kadurin et al., 2012; Wu et al., 2016). This interaction was found to promote the trafficking of the NMDARs to synaptic sites between peripheral dorsal root ganglion neurons and dorsal horn neurons in the spinal cord and is involved in the development of neuropathic pain (Chen et al., 2018). Therefore, it will be of great interest to determine whether fully mature α_2_δ−1 is required for the interaction with BK potassium channels and with NMDARs.

Synchronous stimulated release is often followed by a delayed release occurring after the end of the stimulus, also called asynchronous release (Atluri and Regehr, 1998; Goda and Stevens, 1994; Kaeser and Regehr, 2014). Asynchronous release is thought to be activated by residual Ca^2+^ remaining in the presynaptic terminal after the stimulation (Atluri and Regehr, 1998; Cummings et al., 1996). Although the source of Ca^2+^ responsible for the initiation of synchronous release is indisputably identified from many studies as voltage-gated calcium channels within the active zone (Catterall and Few, 2008; Dolphin, 2012; Nakamura et al., 2015; Zamponi et al., 2015), the source of Ca^2+^ involved in asynchronous release remains uncertain. To study asynchronous release in this work, we took advantage of the optical method developed previously (Ariel and Ryan, 2010) to monitor the slow increase of fluorescence of pHluorin tagged to vGlut-1 after the end of the high frequency stimulation (20 AP at 100 Hz). We show that asynchronous release is reduced in hippocampal presynaptic terminals when uncleaved α_2_δ−1 (α_2_(3C)δ−1) is expressed, and this inhibitory effect is abolished when 3C-protease is co-expressed. Together with our previous report showing that the proteolytic cleavage of α_2_δ is critical for the functional trafficking of Ca_V_2.2 channels to the presynaptic terminals (Kadurin et al., 2016), our data demonstrate that asynchronous release is mediated by Ca^2+^ influx generated by Ca_V_ localized at the presynaptic terminals. Relevant to our data, a study has characterized an asynchronous Ca^2+^ current, recorded after the end of the stimulation pulse, conducted by both Ca_V_2.1 and Ca_V_2.2 channels and activated by the increase of intracellular Ca^2+^ generated by the activity of these channels (Few et al., 2012). This asynchronous current was also identified in mouse hippocampal neurons and this led the authors to suggest that the asynchronous current could contribute to asynchronous release (Few et al., 2012). Other Ca^2+^ sources for asynchronous release have been proposed (Kaeser and Regehr, 2014). Ca^2+^-permeable P2X2 ATP receptors have been involved in asynchronous release in excitatory synapses between CA3 neurons and interneurons in the CA1 region in the hippocampus (Khakh, 2009). At these synapses, P2X2 receptors would be activated by ATP released from vesicles in presynaptic terminals. Further pharmacological characterization would be needed to ascertain the involvement of P2X2 receptors in the asynchronous release we are monitoring in our experimental model. Additionally, in the nucleus of the solitary tract, TRPV1 channels had been suggested to be a source of Ca^2+^ for asynchronous release at excitatory synapses from unmyelinated cranial visceral primary afferent neurons (Peters et al., 2010). However, recent data from the same group have suggested instead that the Ca^2+^ source for asynchronous release would originate from spill-over of intracellular Ca^2+^ from Ca^2+^ nanodomains created by Ca_V_2 channels (Fawley et al., 2016). This latter hypothesis would fit well with our data showing that mature α_2_δ−1 is needed to traffic Ca_V_ to the presynaptic terminals to modulate asynchronous release.

Building on our previous report, we show here that the maturation of α_2_δ is crucial for Ca_V_ channels to fulfil their functional role on synaptic transmission. As α_2_δ−1 expression is upregulated during chronic pain and increases presynaptic Ca_V_2 trafficking (Bauer et al., 2009; Kadurin et al., 2016; Patel et al., 2013; Zamponi et al., 2015), α_2_δ−1 represents a therapeutic target (Zamponi, 2016), and an important question to address for future studies will be to identify endogenous protease(s) involved in the proteolytic maturation of α_2_δ proteins.

## Materials and methods

### Neuronal culture and transfection

All experiments were performed in accordance with the Home Office Animals (Scientific procedures) Act 1986, UK, using a Schedule one method. Hippocampal neurons were obtained from male P0 Sprague Dawley rat pups as previously described (Hoppa et al., 2012). Approximately 75 × 10^3^ cells in 200 μl of plating medium (MEM (Thermo Fisher Scientific) supplemented with B27 (Thermo Fisher Scientific, 2%), glucose (Sigma, 5 mg/ml), transferrin (Millipore, 100 μg/ml), insulin (Sigma, 24 μg/ml), fetal bovine serum (Thermo Fisher Scientific, 10%), GlutaMax (Thermo Fisher Scientific,1%)) were seeded onto sterile poly-L-ornithine-coated glass coverslips. After 24 hr, the plating medium was replaced with feeding medium (MEM supplemented with B27 (2%), glucose (5 mg/ml), transferrin (100 μg/ml), insulin (24 μg/ml), Fetal bovine serum (5%), GlutaMax (1%) and cytosine arabinose (Sigma, 0.4 μM)) half of which was replaced every 7 days. At 7 days in vitro (DIV) and 2 hr before transfection, half of the medium was removed, and kept as ‘conditioned’ medium, and fresh medium was added. The hippocampal cell cultures were then transfected with mCherry, vGlut-pHluorin and either empty vector or α_2_(3C)δ−1 or α_2_(3C)δ−1 + 3C-protease (all cloned in pCAGGs) using Lipofectamine 2000 (Thermo Fisher scientific). After 2 hr, the transfection mixes were replaced with feeding medium consisting of 50% ‘conditioned’ and 50% fresh medium.

### Co-Immunoprecipitation

The protocol was adapted from a procedure described previously (Gurnett et al., 1997).

Briefly, a tsA-201 cell pellet derived from one confluent 75 cm^2^ flask was resuspended in co-IP buffer (20 mM HEPES (pH 7.4), 300 mM NaCl, 1% Digitonin and PI), sonicated for 8 s at 20 kHz and rotated for 1 hr at 4°C. The samples were then diluted with an equal volume of 20 mM HEPES (pH 7.4), 300 mM NaCl with PI (to 0.5% final concentration of Digitonin), mixed by pipetting and centrifuged at 20,000 x g for 20 min. The supernatants were collected and assayed for total protein (Bradford assay; Biorad). 1 mg of total protein was adjusted to 2 mg/ml with co-IP buffer and incubated overnight at 4°C with anti-GFP polyclonal antibody (1:200; BD Biosciences). 30 μl A/G PLUS Agarose slurry (Santa Cruz) was added to each tube and further rotated for 2 hr at 4°C. The beads were then washed three times with co-IP buffer containing 0.2% Digitonin and deglycosylated as previously described alongside with aliquots of the initial WCL prior to co-IP. Laemmli buffer with 100 mM DTT was added to 1 x final concentration and samples were analysed by SDS-PAGE and western blotting with the indicated antibodies as described previously (Kadurin et al., 2016).

The human embryonic kidney tsA-201 cells were obtained from the European Collection of Authenticated Cell Cultures (# 96121229) and tested to be mycoplasma-free.

### Live cell imaging

Coverslips were mounted in a rapid-switching, laminar-flow perfusion and stimulation chamber (RC-21BRFS, Warner Instruments) on the stage of an epifluorescence microscope (Axiovert 200M, Zeiss). Live cell images were acquired with an Andor iXon+ (model DU-897U-CS0-BV) back-illuminated EMCCD camera using OptoMorph software (Cairn Research, UK). White and 470 nm LEDs served as light sources (Cairn Research, UK). Fluorescence excitation and collection was done through a Zeiss 40 × 1.3 NA Fluar objective using 450/50 nm excitation and 510/50 nm emission and 480 nm dichroic filters (for pHluorin) and a 572/35 nm excitation and low-pass 590 nm emission and 580 nm dichroic filters (for mCherry). Action potentials were evoked by passing 1 ms current pulses via platinum electrodes. Cells were perfused (0.5 ml min^−1^) in a saline solution at 32°C containing (in mM) 119 NaCl, 2.5 KCl, 4 CaCl_2_, 25 HEPES (buffered to pH 7.4), 30 glucose, 10 μM 6-cyano-7-nitroquinoxaline-2,3-dione (CNQX) and 50 μM D,L-2-amino-5-phosphonovaleric acid (AP5, Sigma). NH_4_Cl application was done with this solution in which 50 mM NH_4_Cl was substituted for 50 mM NaCl (buffered to pH 7.4). Images were acquired at 100 Hz over a 512 × 266 pixel area in frame transfer mode (exposure time 7 ms) and analyzed in ImageJ (http://rsb.info.nih.gov/ij) using a custom-written plugin (http://rsb.info.nih.gov/ij/plugins/time-series.html). Regions of interest (ROI, 2 μm diameter circles) were placed around synaptic boutons responding to an electrical stimulation of 100 AP at 10 Hz.

### Analysis

Data are given as mean ± SEM or as box (25–75%) and whiskers (10–90%) plots with mean and median (open squares and solid lines). Statistical comparisons were performed using unpaired Student's t test or one-way ANOVA with Bonferroni post-hoc test, using OriginPro 2016.

## Additional files

Supplementary file 1. Statistical information for Figures 1–3.

Transparent reporting form

### Data availability

All data generated or analysed during this study are included in the manuscript.

## References

[bib1] Ariel P, Hoppa MB, Ryan TA (2012). Intrinsic variability in pv, RRP size, ca(2+) channel repertoire, and presynaptic potentiation in individual synaptic boutons. Frontiers in Synaptic Neuroscience.

[bib2] Ariel P, Ryan TA (2010). Optical mapping of release properties in synapses. Frontiers in Neural Circuits.

[bib3] Ariel P, Ryan TA (2012). New insights into molecular players involved in neurotransmitter release. Physiology.

[bib4] Atluri PP, Regehr WG (1998). Delayed release of neurotransmitter from cerebellar granule cells. The Journal of Neuroscience.

[bib5] Augustin I, Rosenmund C, Südhof TC, Brose N (1999). Munc13-1 is essential for fusion competence of glutamatergic synaptic vesicles. Nature.

[bib6] Bauer CS, Nieto-Rostro M, Rahman W, Tran-Van-Minh A, Ferron L, Douglas L, Kadurin I, Sri Ranjan Y, Fernandez-Alacid L, Millar NS, Dickenson AH, Lujan R, Dolphin AC (2009). The increased trafficking of the calcium channel subunit alpha2delta-1 to presynaptic terminals in neuropathic pain is inhibited by the alpha2delta ligand pregabalin. Journal of Neuroscience.

[bib7] Berkefeld H, Sailer CA, Bildl W, Rohde V, Thumfart JO, Eble S, Klugbauer N, Reisinger E, Bischofberger J, Oliver D, Knaus HG, Schulte U, Fakler B (2006). BKCa-Cav channel complexes mediate rapid and localized Ca2+-activated K+ signaling. Science.

[bib8] Bourdin B, Briot J, Tétreault MP, Sauvé R, Parent L (2017). Negatively charged residues in the first extracellular loop of the L-type Ca_V_1.2 channel anchor the interaction with the Ca_V_α2δ1 auxiliary subunit. Journal of Biological Chemistry.

[bib9] Calloway N, Gouzer G, Xue M, Ryan TA (2015). The active-zone protein Munc13 controls the use-dependence of presynaptic voltage-gated calcium channels. eLife.

[bib10] Cantí C, Nieto-Rostro M, Foucault I, Heblich F, Wratten J, Richards MW, Hendrich J, Douglas L, Page KM, Davies A, Dolphin AC (2005). The metal-ion-dependent adhesion site in the von willebrand factor-A domain of alpha2delta subunits is key to trafficking voltage-gated Ca2+ channels. PNAS.

[bib11] Catterall WA, Few AP (2008). Calcium channel regulation and presynaptic plasticity. Neuron.

[bib12] Chen J, Li L, Chen SR, Chen H, Xie JD, Sirrieh RE, MacLean DM, Zhang Y, Zhou MH, Jayaraman V, Pan HL (2018). The α2δ-1-NMDA receptor complex is critically involved in neuropathic pain development and gabapentin therapeutic actions. Cell Reports.

[bib13] Cummings DD, Wilcox KS, Dichter MA (1996). Calcium-dependent paired-pulse facilitation of miniature EPSC frequency accompanies depression of EPSCs at hippocampal synapses in culture. The Journal of Neuroscience.

[bib14] Davies A, Kadurin I, Alvarez-Laviada A, Douglas L, Nieto-Rostro M, Bauer CS, Pratt WS, Dolphin AC (2010). The alpha2delta subunits of voltage-gated calcium channels form GPI-anchored proteins, a posttranslational modification essential for function. PNAS.

[bib15] de Jong APH, Roggero CM, Ho MR, Wong MY, Brautigam CA, Rizo J, Kaeser PS (2018). RIM C_2_B domains target presynaptic active zone functions to PIP_2_-containing membranes. Neuron.

[bib16] Debanne D, Campanac E, Bialowas A, Carlier E, Alcaraz G (2011). Axon physiology. Physiological Reviews.

[bib17] Deng L, Kaeser PS, Xu W, Südhof TC (2011). RIM proteins activate vesicle priming by reversing autoinhibitory homodimerization of Munc13. Neuron.

[bib18] Dickman DK, Kurshan PT, Schwarz TL (2008). Mutations in a Drosophila alpha2delta voltage-gated calcium channel subunit reveal a crucial synaptic function. Journal of Neuroscience.

[bib19] Dolphin AC (2012). Calcium channel auxiliary α2δ and β subunits: trafficking and one step beyond. Nature Reviews Neuroscience.

[bib20] Ermolyuk YS, Alder FG, Henneberger C, Rusakov DA, Kullmann DM, Volynski KE (2012). Independent regulation of basal neurotransmitter release efficacy by variable Ca²+ influx and bouton size at small central synapses. PLoS Biology.

[bib21] Eroglu C, Allen NJ, Susman MW, O'Rourke NA, Park CY, Ozkan E, Chakraborty C, Mulinyawe SB, Annis DS, Huberman AD, Green EM, Lawler J, Dolmetsch R, Garcia KC, Smith SJ, Luo ZD, Rosenthal A, Mosher DF, Barres BA (2009). Gabapentin receptor alpha2delta-1 is a neuronal thrombospondin receptor responsible for excitatory CNS synaptogenesis. Cell.

[bib22] Fawley JA, Hofmann ME, Andresen MC (2016). Distinct calcium sources support multiple modes of synaptic release from cranial sensory afferents. Journal of Neuroscience.

[bib23] Fernández-Alfonso T, Ryan TA (2006). The efficiency of the synaptic vesicle cycle at central nervous system synapses. Trends in Cell Biology.

[bib24] Few AP, Nanou E, Watari H, Sullivan JM, Scheuer T, Catterall WA (2012). Asynchronous Ca2+ current conducted by voltage-gated Ca2+ (CaV)-2.1 and CaV2.2 channels and its implications for asynchronous neurotransmitter release. PNAS.

[bib25] Flockerzi V, Oeken HJ, Hofmann F, Pelzer D, Cavalié A, Trautwein W (1986). Purified dihydropyridine-binding site from skeletal muscle t-tubules is a functional calcium channel. Nature.

[bib26] Goda Y, Stevens CF (1994). Two components of transmitter release at a central synapse. PNAS.

[bib27] Gurnett CA, Felix R, Campbell KP (1997). Extracellular interaction of the voltage-dependent Ca2+ channel alpha2delta and alpha1 subunits. The Journal of Biological Chemistry.

[bib28] Hendrich J, Van Minh AT, Heblich F, Nieto-Rostro M, Watschinger K, Striessnig J, Wratten J, Davies A, Dolphin AC (2008). Pharmacological disruption of calcium channel trafficking by the alpha2delta ligand gabapentin. PNAS.

[bib29] Hoppa MB, Gouzer G, Armbruster M, Ryan TA (2014). Control and plasticity of the presynaptic action potential waveform at small CNS nerve terminals. Neuron.

[bib30] Hoppa MB, Lana B, Margas W, Dolphin AC, Ryan TA (2012). α2δ expression sets presynaptic calcium channel abundance and release probability. Nature.

[bib31] Johnston J, Forsythe ID, Kopp-Scheinpflug C (2010). Going native: voltage-gated potassium channels controlling neuronal excitability. The Journal of Physiology.

[bib32] Kadurin I, Alvarez-Laviada A, Ng SF, Walker-Gray R, D'Arco M, Fadel MG, Pratt WS, Dolphin AC (2012). Calcium currents are enhanced by α2δ-1 lacking its membrane anchor. Journal of Biological Chemistry.

[bib33] Kadurin I, Ferron L, Rothwell SW, Meyer JO, Douglas LR, Bauer CS, Lana B, Margas W, Alexopoulos O, Nieto-Rostro M, Pratt WS, Dolphin AC (2016). Proteolytic maturation of α_2_δ represents a checkpoint for activation and neuronal trafficking of latent calcium channels. eLife.

[bib34] Kadurin I, Rothwell SW, Lana B, Nieto-Rostro M, Dolphin AC (2017). LRP1 influences trafficking of N-type calcium channels via interaction with the auxiliary α_2_δ-1 subunit. Scientific Reports.

[bib35] Kaeser PS, Deng L, Wang Y, Dulubova I, Liu X, Rizo J, Südhof TC (2011). RIM proteins tether Ca2+ channels to presynaptic active zones via a direct PDZ-domain interaction. Cell.

[bib36] Kaeser PS, Regehr WG (2014). Molecular mechanisms for synchronous, asynchronous, and spontaneous neurotransmitter release. Annual Review of Physiology.

[bib37] Kaeser PS, Regehr WG (2017). The readily releasable pool of synaptic vesicles. Current Opinion in Neurobiology.

[bib38] Khakh BS (2009). ATP-gated P2X receptors on excitatory nerve terminals onto interneurons initiate a form of asynchronous glutamate release. Neuropharmacology.

[bib39] Kurshan PT, Oztan A, Schwarz TL (2009). Presynaptic alpha2delta-3 is required for synaptic morphogenesis independent of its Ca2+-channel functions. Nature Neuroscience.

[bib40] Lana B, Page KM, Kadurin I, Ho S, Nieto-Rostro M, Dolphin AC (2016). Thrombospondin-4 reduces binding affinity of [(3)H]-gabapentin to calcium-channel α2δ-1-subunit but does not interact with α2δ-1 on the cell-surface when co-expressed. Scientific Reports.

[bib41] Liu H, De Waard M, Scott VE, Gurnett CA, Lennon VA, Campbell KP (1996). Identification of three subunits of the high affinity omega-conotoxin MVIIC-sensitive Ca2+ channel. Journal of Biological Chemistry.

[bib42] Müller CS, Haupt A, Bildl W, Schindler J, Knaus HG, Meissner M, Rammner B, Striessnig J, Flockerzi V, Fakler B, Schulte U (2010). Quantitative proteomics of the Cav2 channel nano-environments in the mammalian brain. PNAS.

[bib43] Nakamura Y, Harada H, Kamasawa N, Matsui K, Rothman JS, Shigemoto R, Silver RA, DiGregorio DA, Takahashi T (2015). Nanoscale distribution of presynaptic Ca(2+) channels and its impact on vesicular release during development. Neuron.

[bib44] Patel R, Bauer CS, Nieto-Rostro M, Margas W, Ferron L, Chaggar K, Crews K, Ramirez JD, Bennett DL, Schwartz A, Dickenson AH, Dolphin AC (2013). α2δ-1 gene deletion affects somatosensory neuron function and delays mechanical hypersensitivity in response to peripheral nerve damage. Journal of Neuroscience.

[bib45] Peters JH, McDougall SJ, Fawley JA, Smith SM, Andresen MC (2010). Primary afferent activation of thermosensitive TRPV1 triggers asynchronous glutamate release at central neurons. Neuron.

[bib46] Protti DA, Uchitel OD (1997). P/Q-type calcium channels activate neighboring calcium-dependent potassium channels in mouse motor nerve terminals. Pflügers Archiv European Journal of Physiology.

[bib47] Rizzoli SO, Betz WJ (2005). Synaptic vesicle pools. Nature Reviews Neuroscience.

[bib48] Saheki Y, Bargmann CI (2009). Presynaptic CaV2 calcium channel traffic requires CALF-1 and the alpha(2)delta subunit UNC-36. Nature Neuroscience.

[bib49] Schneggenburger R, Sakaba T, Neher E (2002). Vesicle pools and short-term synaptic depression: lessons from a large synapse. Trends in Neurosciences.

[bib50] Simms BA, Zamponi GW (2014). Neuronal voltage-gated calcium channels: structure, function, and dysfunction. Neuron.

[bib51] Südhof TC (2012). The presynaptic active zone. Neuron.

[bib52] Takahashi M, Catterall WA (1987). Identification of an alpha subunit of dihydropyridine-sensitive brain calcium channels. Science.

[bib53] Tong XJ, López-Soto EJ, Li L, Liu H, Nedelcu D, Lipscombe D, Hu Z, Kaplan JM (2017). Retrograde synaptic inhibition is mediated by α-Neurexin binding to the α2δ subunits of N-Type calcium channels. Neuron.

[bib54] Voglmaier SM, Kam K, Yang H, Fortin DL, Hua Z, Nicoll RA, Edwards RH (2006). Distinct endocytic pathways control the rate and extent of synaptic vesicle protein recycling. Neuron.

[bib55] Witcher DR, De Waard M, Sakamoto J, Franzini-Armstrong C, Pragnell M, Kahl SD, Campbell KP (1993). Subunit identification and reconstitution of the N-type Ca2+ channel complex purified from brain. Science.

[bib56] Wu J, Yan Z, Li Z, Qian X, Lu S, Dong M, Zhou Q, Yan N (2016). Structure of the voltage-gated calcium channel Ca(v)1.1 at 3.6 Å resolution. Nature.

[bib57] Xu J, Xiao N, Xia J (2010). Thrombospondin 1 accelerates synaptogenesis in hippocampal neurons through neuroligin 1. Nature Neuroscience.

[bib58] Yazejian B, DiGregorio DA, Vergara JL, Poage RE, Meriney SD, Grinnell AD (1997). Direct measurements of presynaptic calcium and calcium-activated potassium currents regulating neurotransmitter release at cultured Xenopus nerve-muscle synapses. The Journal of Neuroscience.

[bib59] Zamponi GW, Striessnig J, Koschak A, Dolphin AC (2015). The physiology, pathology, and pharmacology of voltage-gated calcium channels and their future therapeutic potential. Pharmacological Reviews.

[bib60] Zamponi GW (2016). Targeting voltage-gated calcium channels in neurological and psychiatric diseases. Nature Reviews Drug Discovery.

[bib61] Zhang FX, Gadotti VM, Souza IA, Chen L, Zamponi GW (2018). BK potassium channels suppress cavα2δ subunit function to reduce inflammatory and neuropathic pain. Cell Reports.

